# Physical and Competitive Environments and Their Influence on Developmental Experiences in Youth Basketball: A Mixed-Methods Study

**DOI:** 10.3390/sports14030086

**Published:** 2026-02-25

**Authors:** Alexandra Folle, Larissa Fernanda Porto Maciel, Mariana Klauck Beirith, Sandy Dorian Isla Alcoser, Sergio José Ibáñez

**Affiliations:** 1 Center of Health and Sports Sciences, Santa Catarina State University, Florianopolis 88080-350, Brazil; alexandra.folle@udesc.br (A.F.); marianaklauck@outlook.com (M.K.B.); 2 Faculty of Education, National University of San Marcos, Lima 15081, Peru; sislaa@unmsm.edu.pe; 3 Faculty of Sport Sciences, University of Extremadura, 10003 Caceres, Spain

**Keywords:** athlete development, youth sport, psychological development

## Abstract

Physical and competitive environments play an important role in shaping athletes’ psychological development, motivation, and long-term engagement in sport. Guided by the Personal Assets Framework, this study examined basketball athletes’ perceptions of the quality of facilities and material resources across developmental stages and explored how these environments influenced developmental experiences and continuity in youth sport. A mixed-methods sequential explanatory design was employed. Quantitative data were collected from Brazilian basketball athletes aged 18–19 years (*n* = 141), followed by semi-structured interviews with 24 athletes. Distributional differences were explored using Kruskal–Wallis tests and associations using chi-square tests, while qualitative data were analysed through thematic analysis. Results indicated that both public and private gyms were commonly used practice settings; however, private facilities were consistently perceived as offering superior structural conditions and material resources, particularly from early adolescence onward. Although physical environments were largely perceived as non-limiting during childhood, their influence on psychological development, motivation, and perceived developmental opportunities became progressively more salient with age. Overall, the findings highlight the importance of adequate physical and competitive environments as key contextual components of athlete development, suggesting that long-term participation in youth sport is strongly influenced by the contexts in which athletes are embedded.

## 1. Introduction

Sport development is strongly influenced by the environments in which athletes engage, train, and compete throughout their formative years. In countries characterised by marked social and geographic inequalities, such as Brazil, access to sport opportunities is unevenly distributed, shaping not only performance trajectories but also athletes’ motivation, engagement, and continuity in sport [1]. Consequently, athletes’ developmental experiences are closely tied to the physical and competitive environments available during youth sport participation.

In addition to institutional and structural conditions, family environments, particularly parental involvement, play a critical role in shaping children’s engagement in sport. Research consistently shows that children’s interest and sustained participation in sport are positively associated with active parental support, modelling of physically active lifestyles, and logistical involvement, such as providing transportation and facilitating access to training opportunities [2,3,4]. Parents often serve as primary socialising agents who introduce children to diverse sport experiences, expose them to different modalities, and help them attribute value and meaning to sport participation [3,5]. However, when parental support is limited, due to socioeconomic constraints, time availability, or lack of access to structured programmes, children may face additional barriers to entering and remaining in sport [6].

Research in youth sport has consistently shown that participation does not occur in isolation and may result in both positive and negative developmental outcomes, depending on the contexts in which it takes place [7]. While social environments—particularly the behaviours, values, and practices promoted by coaches—play a central role in facilitating positive youth development [8], access to sport programmes extends beyond individual choice. Rather, it is shaped by the availability and quality of material, organisational, and social resources that influence athletes’ experiences, opportunities, and sense of belonging within sport environments [9]. Importantly, exposure to constrained or resource-limited environments may also contribute to the development of adaptive motivational patterns, resilience, and strong personal commitment in some athletes, although such conditions simultaneously highlight structural inequalities in access to sport [10].

To better understand the role of context in athlete development, this study adopts the Personal Assets Framework (PAF) as a guiding conceptual model. According to the PAF, developmental experiences emerge from the dynamic interaction between (a) the types of activities in which athletes engage, (b) the quality of the relationships they form—including family and parental influences—and (c) the suitability of the physical and competitive environments surrounding sport participation [8,9]. This framework underscores the central importance of environmental contexts in shaping both developmental processes and long-term engagement in sport.

Consistent with this perspective, research across different sports—including basketball, volleyball, handball, and futsal—has examined how practice contexts influence athletes’ developmental experiences during youth [9,10,11,12,13,14,15]. In Brazil, evidence suggests that organised sport participation has historically occurred within gyms and private clubs, which often serve as athletes’ initial points of contact with structured training environments [1]. However, these environments vary considerably in terms of structural quality, accessibility, and available resources, with important implications for how young athletes experience sport and perceive their developmental opportunities. Differences in access are often intertwined with family support and socioeconomic conditions, which jointly shape pathways into organised sport [16].

Despite growing interest in youth sport environments, empirical research examining how physical and competitive contexts shape athletes’ developmental experiences remains limited, particularly within non-Western and socially unequal sport systems [9]. Moreover, few studies have adopted mixed-methods approaches to capture athletes’ perceptions of sport environments across different developmental stages, especially in the Brazilian context. Therefore, the purpose of this study was to examine how basketball athletes perceive the physical and competitive environments experienced during their formative years and to explore how these conditions—together with family influences— shaped access to practice, motivation, perceived developmental opportunities, psychological engagement, and continuity in basketball.

## 2. Materials and Methods

### 2.1. Study Design

**Figure 1:**
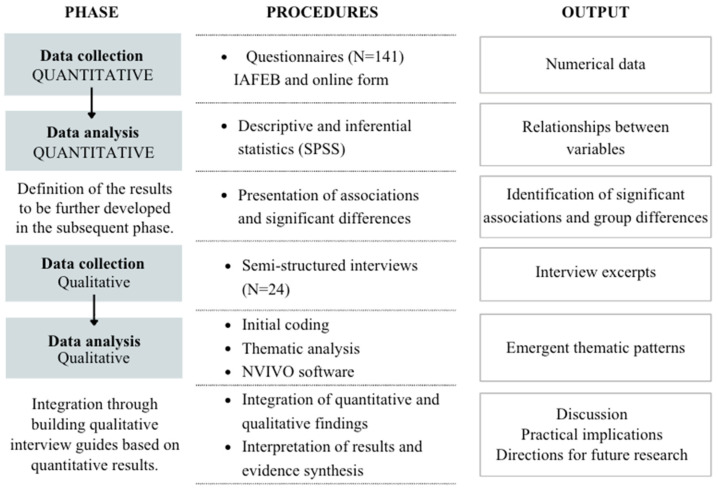
Sequential explanatory mixed-methods design (QUAN → qual). Note: IAFEB = Instrument for the Assessment of Sports Training in Basketball.

This study employed a mixed-methods research (MMR) design using a sequential explanatory strategy (QUAN → qual), grounded in a pragmatic philosophical orientation [17,18]. Following this approach, quantitative data were collected and analysed in Phase 1 to identify patterns in athletes’ perceptions of physical and competitive environments. Subsequently, a qualitative phase (Phase 2) was conducted to explain, deepen, and contextualise the quantitative findings. The integration of quantitative and qualitative methods aimed to provide a comprehensive understanding of how environmental conditions shape basketball athletes’ developmental experiences across their formative years (Figure 1).

### 2.2. Ethical Approval

The study was approved by the Research Ethics Committee for Human Beings of the State University of Santa Catarina, Brazil (Opinion No. 4.733.011). All participants provided written informed consent prior to participation, in accordance with ethical standards for research involving human subjects.

### 2.3. Phase 1: Quantitative Stage

#### 2.3.1. Participants and Sampling

The quantitative phase included 141 Brazilian basketball athletes (78.7% male and 21.3% female) aged 18–19 years. Participants were recruited using a convenience sampling strategy, which is commonly adopted in sport development research involving competitive athletes [19]. This age group was selected because it represents the final youth category prior to transition into adult and professional competitions within the Brazilian basketball system.

Eligibility criteria required participants to (a) be actively involved in organised basketball training and competition and (b) have accumulated experience across multiple developmental stages of youth sport. Recruitment was conducted through a multi-stage online dissemination process. Initially, the study invitation was distributed via email and social media channels of state sport federations and affiliated clubs and was made publicly available on the national federation’s website.

Subsequently, the survey link was shared through social media platforms and online folders created specifically for the study. Additional dissemination occurred through re-sharing by members of the academic community, coaches, athletes, and other sport stakeholders across the country. A non-probability snowball sampling strategy was employed, whereby participants were encouraged to forward the study invitation within their professional and sporting networks [20].

#### 2.3.2. Instrument

Data were collected using the Instrument for the Assessment of Sports Training in Basketball (IAFEB), which was specifically developed and adapted from the Instrument for the Assessment of Sports Training in Volleyball (IAFEV) [21]. The IAFEB is theoretically grounded in the Personal Assets Framework (PAF) [7,8], and the present study focused on the dynamic element related to appropriate physical and competitive contexts.

The instrument is retrospective in nature and organised into sequential age groups corresponding to compulsory stages of Brazilian Basic Education and Higher Education. Section A assessed participation in school-based physical education and consisted of eight items covering four developmental periods: childhood (≤10 years), early adolescence (11–14 years), late adolescence (15–17 years), and early adulthood (18–19 years). Section B assessed engagement in organised sport activities and comprised 46 items, subdivided into four thematic subsections. Participants were asked to report their involvement in these activities according to the specified age periods.

Data were collected online via Google Forms between June and December 2021. Athletes reported their perceptions of (i) the quality of training venues, (ii) the quality of material resources available for basketball practice, and (iii) the perceived influence of physical and competitive environments on their sport development across different developmental stages.

#### 2.3.3. Data Analysis of the Quantitative Phase

Descriptive statistics, including means, standard deviations, and absolute and relative frequencies, were calculated to characterise the sample and summarise response distributions. Data normality was assessed using the Kolmogorov–Smirnov test, which indicated non-normal distributions.

Given these results, non-parametric procedures were adopted. Kruskal–Wallis tests were used to examine differences in athletes’ perceptions across developmental stages, while chi-square (χ^2^) tests were applied to analyse associations between categorical variables. All statistical analyses were performed using SPSS software (version 24.0; SPSS Inc., Chicago, IL, USA), and the level of statistical significance was set at *p* < 0.05.

Because the instrument required participants to retrospectively rate experiences across multiple developmental stages, the dataset includes repeated within-participant observations. The quantitative analyses were conducted primarily for exploratory and descriptive purposes, aiming to identify distributional patterns across developmental stages rather than to support confirmatory causal inferences. Non-parametric tests were therefore interpreted cautiously and in conjunction with the qualitative findings, and the results are presented to identify general distributional trends across stages, and interpretations focus on broad patterns rather than precise estimates of effect magnitude.

### 2.4. Phase 2: Qualitative Stage

The qualitative phase included 24 athletes who were intentionally selected from the quantitative sample using purposive sampling, in accordance with the sequential explanatory mixed-methods design [22]. Selection criteria were established to ensure heterogeneity in developmental trajectories and competitive experiences and included: (i) athletes selected for the Brazilian national team (three men and four women); (ii) athletes selected for state-level teams (four men and three women); and (iii) athletes with the longest involvement in organised basketball (10 men).

This sampling strategy enabled an in-depth exploration of experiences representing different performance levels and regional contexts, thereby supporting a richer interpretation and contextualisation of the quantitative findings.

#### 2.4.1. Data Collection

Semi-structured interviews were conducted using an interview guide developed directly from the main quantitative findings and theoretically informed by the Personal Assets Framework, with particular emphasis on appropriate physical and competitive contexts. The interview guide was organised according to the same sequential age groups used in the IAFEB and explored (i) the quality of training venues, (ii) the availability and adequacy of material resources, and (iii) the structure and organisation of competitive environments across developmental stages.

A pilot interview was conducted to refine question clarity and enhance interviewer communication skills [10]. Participants were contacted using the information provided during Phase 1, with up to four contact attempts made at different times to maximise participation.

Interviews were conducted by telephone between April and July 2022, audio-recorded with participants’ permission, and lasted approximately 60 min on average. All interviews were transcribed verbatim, and transcripts were returned to participants for member checking. Only two participants requested minor amendments, which were incorporated into the final dataset.

#### 2.4.2. Analysis of Data from the Qualitative Phase

Qualitative data were analysed using thematic analysis with a combined deductive–inductive approach [23]. The analysis followed the six established phases: (i) familiarisation with the data; (ii) generation of initial codes using both theory-driven (Personal Assets Framework) and data-driven approaches; (iii) construction of preliminary themes through iterative engagement with the data and visual mapping; (iv) review of themes; (v) definition and refinement of themes; and (vi) writing and interpretation of the analytical narrative.

NVivo software (version 10.2.2; QSR International, Melbourne, Australia) was used to support data management, coding, and analytical transparency. To enhance analytical rigour and reflexivity, a critical friend strategy was adopted, involving regular discussions among members of the research team throughout the analytical process [24].

#### 2.4.3. Integration of Quantitative and Qualitative Data

Integration between quantitative and qualitative strands occurred at both the design and interpretation levels. Qualitative interviews were purposively structured to explain and contextualise key quantitative patterns observed across developmental stages. During analysis, qualitative themes were systematically compared with quantitative trends to generate meta-inferences regarding how athletes interpreted and experienced environmental differences. This integrative process guided the interpretation of results rather than treating the two strands as independent sources of evidence. In line with mixed-methods best practice, this approach also aimed to reduce method-specific bias and strengthen the overall interpretative validity of the study [25].

## 3. Results

Qualitative findings are presented alongside quantitative results to explain and contextualise athletes’ perceptions of physical and competitive environments across developmental stages.

### 3.1. Physical Environments Across Developmental Stages

Quantitative analyses revealed statistically significant distributional differences in athletes’ perceptions of the quality of practice venues across developmental stages (Table 1). Public and private gyms emerged as the most frequently used practice settings throughout athletes’ formative years. Public gyms were predominantly rated as having moderate quality, whereas private gyms were consistently perceived as offering high or very high quality, particularly from early adolescence onwards.

Athletes’ narratives provided contextual depth to these quantitative findings. Public gyms were commonly described as accessible environments, yet structurally limited, often requiring adaptations by athletes and coaches to enable regular training:

*“At the core, the court was open. It wasn’t the best option for us to train, but as it wasn’t anything serious, we could train calmly; there was a normal table, the balls weren’t the best quality, but we could train normally […]”*.

(Athlete 16)

*“[…] it was a cement court. I remember to this day; I was 11 years old when I said to my coach, ‘we train on a pigeon poo court’. We had to clean the court, clean the balls, and then we played on a court where we had to set up all the timers and the scoreboard because it was a rented court […]”*.

(Athlete 8)

From approximately 11 years of age, most athletes reported a transition to more structured environments, primarily private clubs. These settings were characterised by better-maintained courts, greater availability of training spaces, and access to additional resources, such as fitness facilities and specialised staff:

*“The [name of the club] was the best; the gym is gigantic, full of courts, a gym full of equipment, the biggest structure I’ve ever seen and trained in. There were four courts there where you could train basketball and shooting […]”*.

(Athlete 33)

*“The structure was much better than the others in terms of courts; the balls were very well preserved. The tables were maintained almost every month, so they were always good for training. We had a gym available, a fitness coach for pre-training warm-ups, as well as tactical and technical training. It was a much better structure than the others”*.

(Athlete 32)

### 3.2. Availability and Quality of Practice Materials

Consistent with these narratives, quantitative analyses revealed statistically significant distributional differences in athletes’ perceptions of practice materials across developmental stages (Table 2). Before the age of 10 years, training materials were most frequently rated as moderate or low in quality. From early adolescence onwards, materials were increasingly perceived as high or very high quality, particularly among athletes training in private club environments.

Qualitative accounts reinforced these quantitative trends, highlighting material scarcity during early sport initiation:

*“[…] in the beginning, it was those plastic penalty balls. The place was great, the floor was wooden, but we never had help with materials; it was a basket that tore, only the rim was left, the ball was full of pitombo […]”*.

(Athlete 54)

*“[…] what was scarcer were the balls, but for the rest of the materials we found a way to manage, like cones, ropes, things like that.”*

(Athlete 107)

As athletes progressed into more structured training environments, access to higher-quality equipment emerged as a salient feature of their developmental experiences:

*“As it was the club’s subsidiary, it was much better financially. They had more reinforced equipment; the ball was already made of leather, there was a pump, and cones to organise the drills […]”*.

(Athlete 54)

*“[…] it was another world. We arrived, and the first thing we saw in the gym was the wooden court. The club was huge—it was another planet for us. In 2018, I clearly saw the difference between playing in a social project and playing in a club […]”*.

(Athlete 129)

### 3.3. Perceived Influence of Environments on Sport Development

Athletes’ perceptions of the influence of physical environments and material resources on their sport development shifted markedly across developmental stages (Table 3). Up to the age of 10 years, the influence of venues and materials was predominantly perceived as indifferent. From adolescence onwards, this perception changed substantially, with physical and competitive environments increasingly viewed as having a positive or very positive influence on developmental experiences.

This transition was further reflected in athletes’ narratives, which contrasted early experiences marked by adaptation and constraint with later experiences characterised by stability and institutional support:

*“[…] The equipment was enough. Obviously, it could have been improved a lot, even because sometimes there was no court to train on or we had to train at very inconvenient times. There were conditions that allowed us to start, but it was not always ideal. Still, it was enough to get started”*.

(Athlete 19)

*“When it rained a lot, we did not come, but when it rained and then stopped, we were able to train even when the court was wet. When the weather was bad, we took risks, but when it rained heavily, the coach offered online training so we could train at home […]”*.

(Athlete 107)

In contrast, athletes training in well-resourced clubs described environments that actively facilitated their development and reduced contextual barriers:

*“[…] The [name of the club] is totally different from the [name of the project]; it’s another world. Structurally, it’s a fantastic club. You have a court available all day, a great gym inside the club, physiotherapy services, everything […]”*.

(Athlete 102)

*“It was very good because they received a lot of government investment. The courts were excellent, new balls arrived every week, and the gym was sensational. Even before the renovation, the structure was already very good, and the court is one of the best in [name of city] […]”*.

(Athlete 129)

### 3.4. Regional Disparities in Competitive Environments

Analysis of the interview data revealed marked regional differences in athletes’ experiences of competitive environments. Two contrasting patterns emerged: regions with limited access to structured basketball contexts, predominantly in the North and Northeast of Brazil, and regions offering more favourable developmental conditions, mainly in the South and Southeast.

Athletes from less-resourced regions described restricted access to competitions, limited financial support, and inadequate infrastructure:

*“Apart from soccer, you have to prove that the sport is good for people to want to practise it. Here, only schools play, and teams that have money and are really passionate about basketball”*.

(Athlete 64, Amazonas)

*“Basketball should improve a lot here; it is very undervalued in Maranhão […]”*.

(Athlete 46, Maranhão)

*“[…] here there is usually one team per city, but not every city competes because there is no money to travel and no investment from the city. Sometimes even the city’s court is very poor, and there is no way to organise championships. In the end, most teams are concentrated in Salvador”*.

(Athlete 129, Bahia)

Conversely, athletes from the South and Southeast regions reported greater institutional support, better infrastructure, and higher-quality competitive opportunities:

*“[…] the good thing about the state of Paraná is that it strongly encourages athletes. Being called up to a state or national team already brings scholarships. If you make the national team, it’s another level, and participating in national championships is highly valued […]”*.

(Athlete 8, Paraná)

*“[…] here in Santa Catarina there is a lot of support from the local government; they really encourage sport”*.

(Athlete 90, Santa Catarina)

*“[…] this is true for any sport: the main showcases are usually Rio de Janeiro, São Paulo, and Santa Catarina. There is no way to play professionally in some regions, such as Mato Grosso”*.

(Athlete 120, Santa Catarina)

### 3.5. Summary of Integrated Findings

Overall, the integrated findings indicate that basketball athletes’ developmental experiences were strongly shaped by the quality and accessibility of physical and competitive environments. Early sport participation was frequently characterised by limited infrastructure and material resources, requiring adaptation and resilience. As athletes progressed through adolescence, access to more structured environments—primarily private clubs—was associated with more positive developmental perceptions, increased motivation, and greater continuity in sport participation. These experiences varied substantially across Brazilian regions, reflecting broader structural and sociocultural inequalities within the national sport system.

## 4. Discussion

The present study examined how physical and competitive environments experienced during formative years shape Brazilian basketball athletes’ developmental experiences. From a psychological perspective, the findings demonstrate that environmental conditions progressively influence athletes’ motivation, engagement, and decisions to remain involved in sport. Public and private gyms emerged as the primary contexts for participation, with private facilities consistently perceived as offering superior structural conditions and material resources. Beyond purely infrastructural differences, these contrasts reflect underlying economic inequalities that shape athletes’ access to training opportunities and influence how they perceive their possibilities for development within sport.

Although environmental quality and material availability were not perceived as limiting participation during early childhood, this influence was largely viewed as indifferent rather than developmentally optimal. From approximately 11 years of age onward, this perception shifted, coinciding with athletes’ transition to more structured and resource-rich environments. This transition frequently depends on families’ economic capacity to support costs related to transportation, equipment, and programme fees, which may operate as informal selection mechanisms. As a result, economic conditions not only affect access to facilities but also shape athletes’ expectations and perceptions of what constitutes a viable developmental pathway. These results highlight the dynamic role of environmental conditions in shaping motivation, engagement, and continuity in sport across developmental stages [26].

From a developmental perspective, early sport initiation in Brazil frequently occurs in contexts characterised by limited infrastructure and material resources. While athletes retrospectively described these conditions as sufficient to initiate participation, qualitative narratives revealed the need for constant adaptation, improvisation, and resilience. Such experiences may contribute to the development of coping strategies and psychological adaptability; however, they also indicate unequal structural starting points. When economic constraints persist without adequate institutional support, they may restrict access to systematic training and competitive exposure, thereby narrowing developmental opportunities. As athletes mature and developmental demands increase, the adequacy of physical and material environments becomes increasingly salient for perceived progression and long-term engagement.

These findings align with the Personal Assets Framework, which posits that appropriate physical and competitive contexts interact with activities and relationships to shape developmental experiences [7,8,9]. Within this framework, adolescence represents a critical period during which environmental suitability increasingly influences motivation, perceived competence, and sustained participation. The present results suggest that environments lacking adequate structure may limit opportunities for continued engagement, even when early participation is possible. Conversely, coordinated investments in infrastructure, programme organisation, and coaching quality may expand access to meaningful developmental experiences and positively influence how young athletes interpret their sport environments.

Regarding practice settings, the findings reinforce previous evidence indicating that, although organised sport participation in Brazil has historically been centred in private clubs, public programmes and government initiatives play a relevant role in providing initial access to basketball [1,10]. Similar patterns have been observed across different sports, with variations related to gender, competitive level, and institutional organisation [11,12,27]. Together, these findings underscore the complementary roles of public and private contexts in athlete development, while also revealing substantial disparities in quality and accessibility. Strengthening articulation between these sectors may help create more continuous developmental pathways and broaden access to higher-quality training environments.

Despite the importance of public initiatives, the superior quality of private facilities identified in this study appears to drive athletes’ migration toward these environments from early adolescence onwards. This pattern is consistent with evidence demonstrating that age and infrastructure conditions influence athletes’ decisions regarding participation contexts [28]. Comparable trajectories have been reported among elite Brazilian basketball athletes, who often relocate within or across states in search of improved training conditions [10]. However, access to such environments is rarely determined by sport-related factors alone and is strongly mediated by economic resources that enable families to sustain long-term investment in sport.

The pursuit of and permanence in well-structured environments are closely linked to athletes’ socio-economic conditions. Although narratives of upward social mobility through sport are common, evidence indicates that high-performance athletes disproportionately originate from middle-class families, who are better positioned to access private clubs and absorb the financial costs associated with long-term participation [9]. This pattern is consistent with national data showing a positive association between income and engagement in physical activity in Brazil [29]. Although socio-economic variables were not quantitatively assessed in the present study, their prominence in athletes’ narratives highlights their relevance for understanding developmental inequalities. Initiatives such as financial assistance programmes, transportation support, and inclusive talent identification systems may help reduce these disparities and expand access to environments that foster development.

Regional disparities further illustrated how broader structural and sociocultural contexts shape developmental opportunities. Athletes from the South and Southeast regions described more favourable sport ecosystems, characterised by institutional support, financial incentives, and regular competitive opportunities. These findings corroborate previous research highlighting the prominence of these regions in Brazilian basketball development [10,11,26,30]. In contrast, athletes from the North and Northeast reported limited access to competitions, infrastructure, and funding, constraining developmental pathways and reinforcing regional inequalities within the national sport system. Addressing these asymmetries requires coordinated policy efforts aimed at redistributing resources and expanding competitive opportunities across regions.

The extreme distributions observed in the overall impact variable should also be interpreted with caution. Because the instrument relied on retrospective self-report across multiple developmental stages, these patterns may partially reflect recall framing and instrument effects, in addition to genuine developmental differences. Younger childhood experiences may be remembered in more global or affectively neutral terms, whereas later stages are often recalled with greater evaluative specificity. This methodological characteristic highlights the importance of interpreting the quantitative findings in conjunction with the qualitative narratives.

Collectively, these findings demonstrate that athlete development is not solely the result of individual talent or effort but is deeply embedded within environmental, structural, and socio-economic contexts. The quality and accessibility of physical and competitive environments shape not only technical development but also athletes’ motivation, sense of belonging, and decisions to persist in sport. From a psychological perspective, these results underscore the importance of sport environments in shaping experiences relevant to psychological well-being and adaptative functioning in sport. They also suggest that strategic investments in inclusive sport systems can transform structural constraints into opportunities, positively influencing how young athletes interpret their developmental trajectories.

Taken together, the mixed-methods findings generate meta-inferences regarding the developmental role of sport environments. The quantitative patterns identify stage-related differences in perceived environmental influence, while the qualitative narratives explain how athletes interpret and assign meaning to these contexts. This integration suggests that environmental quality shapes not only access to resources but also athletes’ evolving perceptions of opportunity, belonging, and developmental possibility.

## 5. Limitations and Practical Implications

This study has some limitations that should be acknowledged. The retrospective nature of the quantitative instrument relies on athletes’ recall of past experiences, which may be subject to memory bias. An additional limitation concerns the retrospective repeated-measures structure of the quantitative data. Although non-parametric tests were used to explore distributional patterns, the dependence between observations limits the strength of inferential conclusions. Therefore, statistical findings should be interpreted as indicative trends rather than confirmatory evidence. However, the mixed-methods design and the integration of qualitative narratives contributed to contextualising and deepening the interpretation of these perceptions.

In addition, the sample was limited to Brazilian basketball athletes aged 18–19 years, which may restrict the generalisability of the findings to other age groups, sports, or national contexts. Although socio-economic factors and family support emerged as relevant influences in athletes’ narratives, these variables were not directly measured and should be addressed more explicitly in future research through the inclusion of objective economic indicators and longitudinal designs.

An additional limitation concerns the absence of effect size estimates and post hoc comparisons. Given the exploratory and retrospective nature of the quantitative design, the analyses were intended to describe broad distributional patterns rather than provide precise inferential estimates. Future research using prospective designs and repeated-measures modelling could extend these findings by examining effect magnitudes and stage-specific contrasts.

Despite these limitations, the findings offer relevant practical implications for youth sport development. The results highlight the importance of providing adequate physical and material environments, particularly during adolescence, when environmental conditions appear to play a more decisive role in motivation, engagement, and continuity in sport. Investment in the quality and maintenance of public sport facilities may help reduce inequalities in access and support sustained participation. Establishing partnerships between schools, municipalities, and sport organisations may expand opportunities for structured training and competitive exposure.

Coaches and sport organisations should recognise that while early sport participation can occur in modest environments, long-term development requires progressive access to structured and resource-rich contexts. Creating clearer developmental pathways between public programmes and club-based systems, including scholarship schemes and support mechanisms for economically disadvantaged athletes, may facilitate retention and promote more equitable access. Finally, policymakers and sport administrators should account for regional disparities when designing youth sport policies, with targeted investment in less-resourced regions to promote more balanced developmental ecosystems and positive long-term sport experiences.

## 6. Conclusions

This study demonstrates that basketball athletes’ developmental experiences are strongly shaped by the physical and competitive environments encountered throughout youth sport participation. While early engagement in basketball often occurred in contexts characterised by limited infrastructure and material resources, access to more structured and better-resourced environments during adolescence emerged as a key factor influencing athletes’ perceptions of development, motivation, and continuity in sport. These findings reinforce that athlete development involves fundamentally psychological processes, embedded within physical, social, and structural contexts.

Guided by the Personal Assets Framework, the results highlight that appropriate physical and competitive environments interact with activities and relationships to support positive developmental experiences. In unequal sport systems such as Brazil, disparities in environmental quality and accessibility contribute to differentiated developmental pathways, underscoring the importance of contextual factors beyond individual characteristics.

From an applied perspective, the findings emphasise the need for youth sport systems that provide not only initial access to participation but also progressive environmental conditions capable of sustaining engagement over time. Addressing structural and regional inequalities in sport environments may enhance developmental experiences, support long-term participation, and contribute to more equitable and psychologically supportive pathways in youth sport, with potential implications for psychological well-being and athlete’s subjective sport experiences. These findings contribute to a more context-sensitive understanding of youth sport development in socially unequal settings.

## Figures and Tables

**Table 1:** Perceived quality of physical and competitive practice environments across developmental stages.

Practice Settings	Quality	≤10 Years		11–14 Years		15–17 Years		18–19 Years		χ^2^ (*p*)
Public Places	Low quality	47	(43%)	37	(34%)	2	(18%)	24	(33%)	9.65 (*p* = 0.646)
	Moderate	42	(39%)	44	(40%)	7	(64%)	36	(49%)	
	High quality	19	(18%)	28	(26%)	2	(18%)	13	(18%)	
Public Gym	Low quality	29	(29%)	27	(25%)	4	(26%)	27	(39%)	25.91 (*p* = 0.014)
	Moderate	51	(50%)	48	(46%)	1	(7%)	21	(30%)	
	High quality	22	(22%)	19	(27%)	10	(66%)	21	(31%)	
Private Gym	Low quality	26	(23%)	12	(9%)	2	(6%)	15	(17%)	43.92 (*p* < 0.001)
	Moderate	15	(14%)	11	(9%)	6	(18%)	22	(26%)	
	High quality	69	(63%)	104	(82%)	26	(76%)	49	(57%)	
School	Low quality	48	(48%)	34	(31%)	4	(33%)	20	(27%)	19.50 (*p* = 0.077)
	Moderate	31	(31%)	36	(33%)	6	(50%)	24	(33%)	
	High quality	21	(21%)	39	(35%)	2	(16%)	29	(40%)	
Alternative settings	Low quality	38	(64%)	29	(46%)	1	(25%)	21	(47%)	17.35 (*p* = 0.137)
	Moderate	18	(30%)	19	(30%)	3	(75%)	16	(36%)	
	High quality	3	(5%)	14	(23%)	0	(0%)	7	(15%)	

Note. Percentages are calculated within stage-specific valid responses. The number of valid responses varies across developmental stages due to missing retrospective data. Observations represent repeated ratings from the same participants. χ^2^ = chi-square test; *p* = significance level.

**Table 2:** Perceived quality of materials for basketball practice across developmental stages.

Materials	Quality	≤10 Years		11–14 Years		15–17 Years		18–19 Years		χ^2^ (*p*)
Balls, cones, tables	Low quality	17	(12%)	10	(7%)	0	(0%)	4	(4%)	139.8 (*p* < 0.001)
	Moderate	61	(44%)	17	(12%)	4	(10%)	43	(44%)	
	High quality	59	(43%)	112	(81%)	34	(89%)	51	(52%)	
Uniforms, vests	Low quality	33	(24%)	11	(8%)	0	(0%)	10	(10%)	192.3 (*p* < 0.001)
	Moderate	51	(37%)	16	(11%)	0	(0%)	49	(50%)	
	High quality	53	(39%)	112	(81%)	38	(100%)	39	(40%)	

Note. Percentages are calculated within stage-specific valid responses. The number of valid responses varies across developmental stages due to missing retrospective data. Observations represent repeated ratings from the same participants. χ^2^ = chi-square test; *p* = significance level.

**Table 3:** Influence of location and practice materials on sports development in basketball.

Influence of Location and Materials	Quality	≤10 Years		11–14 Years		15–17 Years		18–19 Years		χ^2^ (*p*)
Overall impact	Negative	3	(2%)	9	(6%)	0	(0%)	0	(0%)	648.5 (*p* < 0.001)
	Indifferent	124	(90%)	1	(1%)	0	(0%)	1	(1%)	
	Positive	10	(7%)	129	(93%)	38	(100%)	97	(99%)	

Note. Percentages are calculated within stage-specific valid responses. The number of valid responses varies across developmental stages due to missing retrospective data. Observations represent repeated ratings from the same participants. χ^2^ = chi-square test; *p* = significance level.

## Data Availability

The datasets employed in this study can be obtained from the corresponding author upon reasonable request. However, certain data cannot be made publicly accessible due to privacy considerations.
